# Uric acid to albumin ratio as a novel predictor for coronary slow flow phenomenon in patients with chronic coronary syndrome and non-obstructive coronary arteries

**DOI:** 10.1186/s12872-024-04040-5

**Published:** 2024-07-13

**Authors:** Xiao-jiao Zhang, Ai-jie Hou, Bo Luan, Cheng-fu Wang, Jia-jin Li

**Affiliations:** Department of Cardiology, The People’s Hospital of Liaoning Province, The People’s Hospital of China Medical University, Shenyang, China

**Keywords:** Uric acid to albumin ratio, Coronary slow flow phenomenon, Chronic coronary syndrome, Predictors, Coronary artery disease

## Abstract

**Background:**

The plasma uric acid to albumin ratio (UAR) is considered as a novel indicator for Inflammation. However, the association between UAR and coronary slow flow phenomenon (CSFP) remains unclear.

**Methods:**

A total of 1328 individuals with chronic coronary syndrome (CCS) receiving coronary angiography (CAG) and found no obvious obstructive stenosis (< 40%) were included in this study. 79 individuals developed CSFP and were divided into CSFP group. The 1:2 age-matched patients with normal coronary blood flow were allocated to the control group (*n* = 158). The clinical characteristics, laboratory parameters including uric acid, albumin ratio, UAR and the angiographic characteristics were compared between the two groups.

**Results:**

Patients with CSFP had a higher level of uric acid (392.3 ± 85.3 vs. 273.8 ± 71.5, *P* < 0.001), UAR (10.7 ± 2.2 vs. 7.2 ± 1.9, *P* < 0.001), but a lower level of plasma albumin (36.9 ± 4.2 vs. 38.5 ± 3.6, *P* = 0.003). Moreover, UAR increased as the numbers of vessels involved in CSFP increased. The logistic regression analysis demonstrated that UAR was independent predictors for CSFP. The Receiver operating characteristic (ROC) curve analysis showed that when UAR was more than 7.9, the AUC was 0.883 (95% CI: 0.840–0.927, *p* < 0.001), with the sensitivity and specificity were 78.2% and 88.2% respectively.

**Conclusion:**

Combined uric acid with plasma albumin, UAR could serve as an independent predictor for CSFP.

## Introduction

Coronary slow flow phenomenon (CSFP) is defined as slow coronary blood flow in the three main coronary arteries without obstructive lesions (< 40%) [1]. The TIMI frame count (TFC) [1] were used to quantitatively evaluate the coronary blood flow of the three coronary arteries. Different from traditional classic coronary atherosclerotic heart disease, CSFP had a unique and different pathophysiology as well as clinical outcome [2]. Although accumulating evidences have suggested that CSFP was associated with inflammation [2–4], subclinical atherosclerosis [5], oxidative stress [4], and endothelial dysfunction [4, 6]. However, the underlying pathogenesis for CSFP remains unknown. It is estimated that nearly 80% of the patients with CSFP suffered recurrent resting angina pectoris, and 20% of the CSFP patients were readmitted to emergency room or CCU, which greatly affected the quality of life in these patients [6].

As the end product of purine derivatives, serum uric acid, has been demonstrated to play a key role in vascular damage, both in in vitro and in vivo studies [7, 8]. The serum uric acid has also been suggested as a risk factor for atherosclerosis, which bring in the occurrence and development of coronary artery disease (CAD) [9, 10]. Moreover, an elevated level of uric acid is demonstrated to associate with endothelial dysfunction and increased chronic inflammatory response [11, 12]. As the most abundant protein in human body, the serum albumin plays an important role in anti-inflammatory [13] and eliminating free radicals [14], which could bring in a protective effect for endothelium cell. Recently, uric acid to albumin ratio (UAR) has been suggested as a novel predictive indicator for mortality in patients with ST segment elevation myocardial infarction (STEMI) [15], as well as non-ST segment elevation myocardial infarction (NSTEMI) [16], and unstable angina pectoris [17]. However, the relationship between UAR and CSFP has not been well evaluated in the literature. Since the UAR have the similar pathophysiological basis with CSFP. So in this study, we aimed to explore the potential relationship between UAR and CSFP, so as to improve the management of these patients.

## Methods

### Study population

**Fig. 1:**
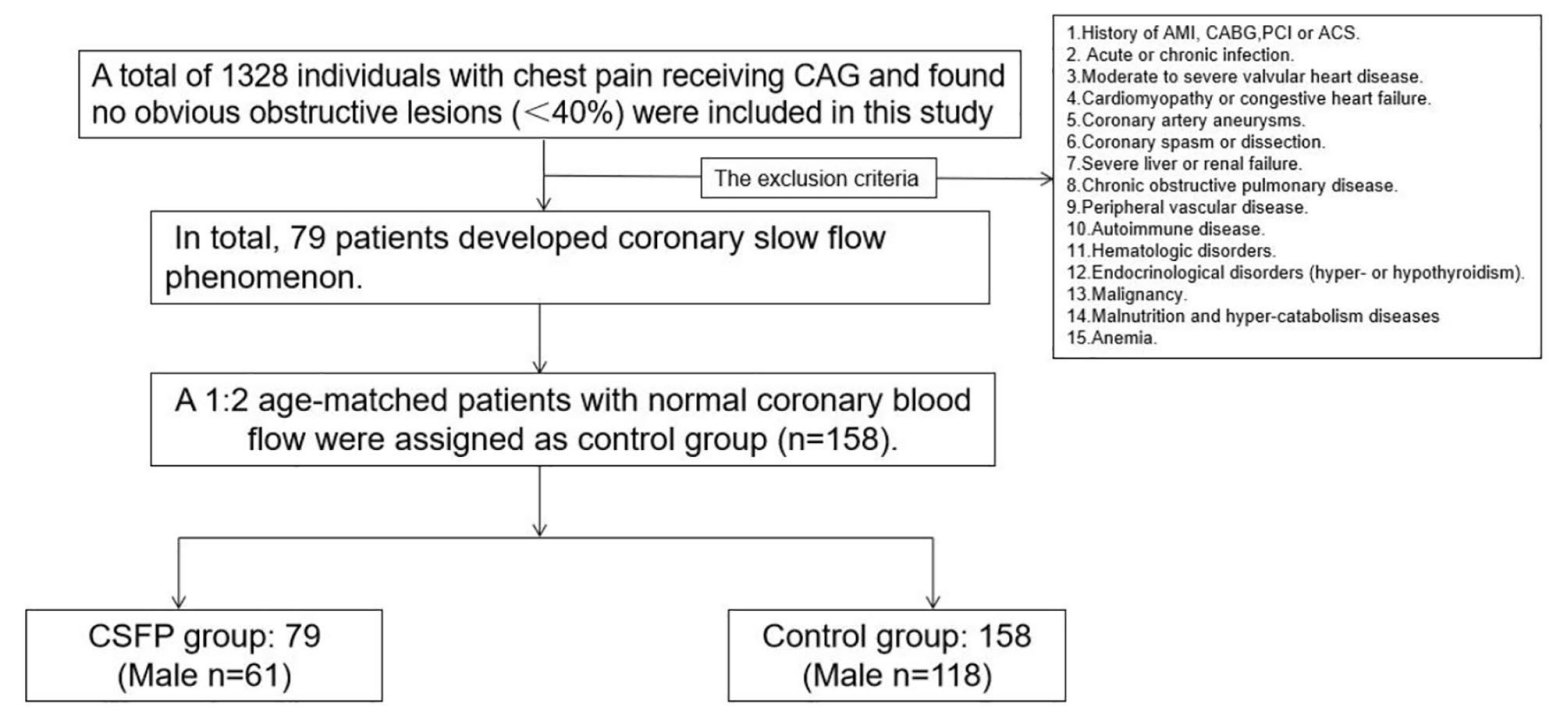
The study flow chart

This is a single center prospective cohort study.The study flow chat was showed in Fig. 1. A total of 1328 individuals with chest pain and chronic coronary syndrome (CCS) receiving CAG and found no obvious obstructive lesions (< 40%) were included in this study from March 2019 to March 2023. The individuals included were then divided into CSFP group and the control group according to the angiographic coronary blood flow. The exclusion criteria were displayed in Fig. 1. At last, 79 individuals were diagnosed as CSFP, which were assigned to the CSFP group. Then the 1:2 age-matched patients with normal coronary blood flow were allocated to the control group (*n* = 158). Eventually, 237 patients were included in this study.

### Laboratory measurements and definitions

The blood samples were collected after a night of fasting and then sent to the central laboratory of our hospital for testing within a hour. The routine blood biochemical examination including fasting blood glucose, blood lipids, liver and kidney function were detected in all of the patients included. UAR was calculated as uric acid divided by plasma albumin. The parameters were compared between the two groups.

### Coronary angiography

The right radial was the most preferred access for coronary angiography (CAG). The procedure was performed by the experienced interventional cardiologists with the standard Judkins technique. The TIMI frame count (TFC) [2] were used to quantitatively evaluate the coronary blood flow of the three coronary arteries including the left anterior descending artery (LAD), left circumflex artery (LCX), right coronary artery (RCA), which was calculated as the last frame minus the first frame. The first frame was defined as > 70% lumen filling with an antegrade contrast agent, and the last frame was determined as the antegrade contrast agent filling to a certain distal landmark for different vessels. The distal bifurcation (“whale’s tail”), distal bifurcation of the obtuse marginal branch, and the first branch of the posterolateral artery were used as the distal landmark for the LAD, LCX, RCA, respectively. As the LAD is much longer than other arteries, so the corrected TFC (TFC divided by 1.7) was developed [2]. In the literature, the most widely used diagnostic threshold of TFC were 36.2 ± 2.6 for the LAD (21.1 ± 1.5 cTFC for LAD), 22.2 ± 4.1 for the LCX, and 20.4 ± 3 for the RCA [2]. Patients were diagnosed as CSFP when TFC in any of the coronaries were over the threshold above.

### Statistical analysis

The SPSS 22.0 was used for data analysis. The categorical data were displayed as rates or percentages, which were compared using chi-square or the Fisher exact test. The continuous variables were expressed as the mean ± standard deviation or median, as applicable, which were assessed using Student’s t-test and the Mann-Whitney U test. The univariable regression analysis was performed to investigate the factors related to CSFP and logistic regression analysis was carried out to explore the independent predictors for CSFP. The receiver operating characteristic (ROC) curve analysis was used to determine the diagnostic value of the factors determined. A 2-sided *P* < 0.05 was considered statistical difference.

## Results

### Baseline and clinical characteristics

The baseline demographic characteristics, clinical comorbidity and pre-procedural medication history were shown in Table 1. The incidence of male and diabetes mellitus were comparable between the two groups. Also, there were no difference in the blood pressure and medication history except for nitrates (*P* > 0.05). However, the proportion of current smokers and hypertension in CSFP group were significantly higher than in the controls. Moreover, patients with CSFP tended to use nitrates more often. (*P* < 0.05) (Table 1).

**Table 1:** Baseline characteristics and medication of the two groups

	CSFP group (*n*=79)	Control group (*n*=158)	*P* value
Age, years	61.2 ± 9.6	61.2 ± 9.6	1
Male sex, n (%)	61(77.2)	118(74.7)	0.750
BMI, Kg/m^2^	25.0 ± 2.7	24.9 ± 3.2	0.779
Systolic blood pressure, mmHg	122.8 ± 17.8	125.6 ± 15.9	0.227
Diastolic pressure, mmHg	76.2 ± 10.5	76.2 ± 10.6	1
Current smoking, n (%)	33(41.8)	44(27.8)	0.039
Hypertension, n (%)	50(63.3)	68(43.0)	0.004
Diabetes mellitus, n (%)	25(31.6)	38(24.1)	0.217
ACEI/ARB/ARNI, n (%)	20(25.3)	36(22.8)	0.746
Beta-blocker, n (%)	19(24.1)	32(20.3)	0.507
Calcium canal blocker, n (%)	19(24.1)	33(20.9)	0.619
Antiplatelet, n (%)	26(32.9)	49(31.0)	0.769
Statin, n (%)	28(35.4)	52(32.9)	0.771
Nitrates, n (%)	25(31.6)	30(19.0)	0.034

BMI: body mass index, ACEI: angiotensin-converting enzyme inhibitor, ARB: angiotensin II receptor blocker, ARNI: angiotensin receptor enkephalinase inhibitor

### Laboratory parameters of the two groups

**Fig. 2:**
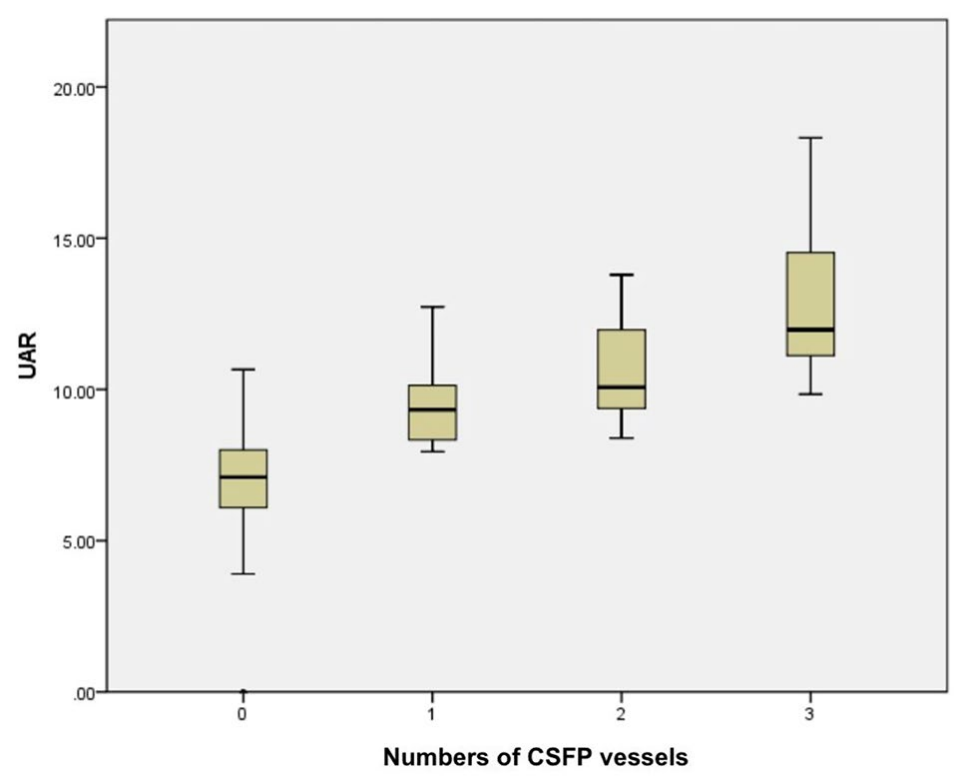
Correlation between the number of vessels involved in CSFP and UAR

The laboratory parameters are displayed in Table 2. There were no statistical differences between the two groups with regard to fasting glucose, estimated glomerular filtration rate (eGFR), total cholesterol, triglyceride, high-density lipoprotein cholesterol (HDL-C) and low-density lipoprotein cholesterol (LDL-C). However, patients with CSFP tended to suffer a higher level of uric acid (392.3 ± 85.3 vs. 273.8 ± 71.5, *P* < 0.001), and UAR (10.7 ± 2.2 vs. 7.2 ± 1.9, *P* < 0.001), but a lower level of plasma albumin (36.9 ± 4.2 vs. 38.5 ± 3.6, *P* = 0.003). (Table 2). Moreover, UAR increased as the numbers of vessels involved in CSFP increased, which had statistical significance. (Fig. 2).

**Table 2:** Laboratory parameters of the two groups

	CSFP group (*n*=79)	Control group (*n*=158)	*P* value
Fasting glucose, mmol/l	5.7 ± 1.6	5.9 ± 2.0	0.501
eGFR, ml/min	70.4 ± 15.3	71.9 ± 14.4	0.469
Uric acid, umol/L	392.3 ± 85.3	273.8 ± 71.5	< 0.001
Albumin, g/L	36.9 ± 4.2	38.5 ± 3.6	0.003
Total cholesterol, mmol/l	4.3 ± 1.1	4.2 ± 1.0	0.273
Triglyceride, mmol/l	1.8 ± 0.9	1.8 ± 1.1	0.756
HDL-C, mmol/l	1.0 ± 0.3	1.0 ± 0.3	0.155
LDL-C, mmol/l	2.8 ± 0.9	2.6 ± 0.9	0.122
UAR	10.7 ± 2.2	7.2 ± 1.9	< 0.001

eGFR: estimated glomerular filtration rate, HDL-C: high-density lipoprotein cholesterol, LDL-C: low-density lipoprotein cholesterol, UAR: uric acid to albumin ratio

### Angiographic characteristics of the two groups

The characteristics of the vessels involved in ths CSFP were shown in Table 3. Patients with CSFP had a higher TFC, cTFC or the mean TFC than the controls. (*P* < 0.001). From the aspect of the coronary arteries affected, the LAD (65.8%) and RCA (65.8%) were the most common vessels, while the least common artery was LCX (65.8%). In terms of the numbers of vessels involved in the CSFP, the single vessel affected came the first (40.5%), followed by two vessels (32.9%), and the last were three vessels (26.6%).(Table 3).

**Table 3:** Angiographic characteristics of the two groups

	CSFP group (*n*=79)	Control group (*n*=158)	*P* value
TIMI frame count			< 0.001
LAD	26.9 ± 4.2	20.9 ± 1.8	
LCX	24.4 ± 4.7	19.2 ± 2.4	
RCA	25.7 ± 5.2	19.4 ± 2.4	
mean TFC	25.7 ± 2.8	19.8 ± 1.4	
Distribution of CSFP			
LAD, n (%)	52(65.8)		
LCX, n (%)	43(54.4)		
RCA, n (%)	52(65.8)		
Numbers of vessels involved in CSFP 1, n (%) 2, n (%) 3, n (%)	32(40.5) 26(32.9) 21(26.6)		

TIMI: thrombolysis in myocardial infarction, LAD: left anterior descending artery, LCX: left circumflex artery, RCA: right coronary artery, TFC: TIMI frame count, CSFP: coronary slow flow phenomenon

### Independent predictors for CSFP

**Fig. 3:**
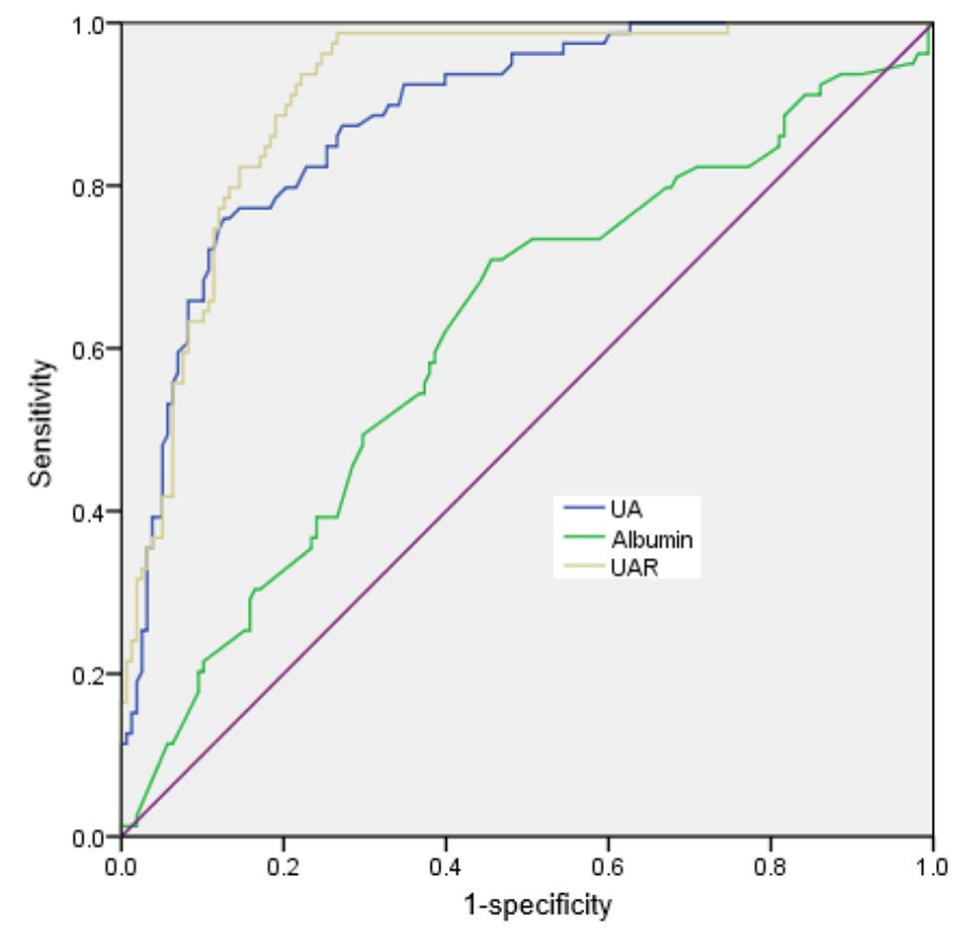
ROC curve showing the predicting value of risk factors for the presence of CSFP

The univariate analysis was used to investigate the potential factors associated with CSFP. We discovered that CSFP was related to hypertension, smoking and UAR. (*p* < 0.05). The logistics analysis showed that UAR was independent predictor for CSFP. (*p* < 0.05) (Table 4). The receiver operating curve (ROC) revealed that when uric acid was ≥ 347.5mmol/L, the area under the curve (AUC) was 0.846 (95% CI: 0.815–0.918, *p* < 0.001) with the sensitivity and specificity were 75.9% and 86.3%, respectively. When plasma albumin was less than 38.5 g/L, the AUC was 0.667 (95% CI: 0.541–0.764, *p* = 0.003), with the sensitivity and specificity were 70.9% and 62.8%, respectively. When UAR was more than 7.9, the AUC was 0.883 (95% CI: 0.840–0.927, *p* < 0.001), with the sensitivity and specificity were 78.2% and, 88.2% respectively. (Fig. 3). As a new indicator, combined uric acid with plasma albumin, UAR could significantly increase the predictive value for the presence of CSFP.

**Table 4:** Univariate and multivariate logistic regression analysis for presence of CSFP.

	Univariate analysis			Multivariate analysis		
	OR	95% CI	P值	OR	95% CI	P值
Hypertension	1.590	1.271–3.348	0.027	1.552	0.737–3.343	0.325
Current smoker	2.040	1.354–4.973	0.017	1.923	0.837–4.973	0.265
UAR	1.826	1.223–9.215	0.030	1.822	1.219–9.227	0.028

UAR: uric acid to albumin ratio

## Discussion

In this study, we firstly investigated the relationship between UAR and CSFP, and discovered that patients with CSFP tended to have an elevated level of UAR than the controls. Moreover, the UAR elevated as the numbers of vessels involved in the CSFP increased. Furthermore, multivariate logistic regression analysis demonstrated that UAR was an independent predictor for CSFP, which showed a better predictive value than uric acid or albumin alone. As far as we know, this is the firs study to explore the potential role of UAR for the pathophysiology of CSFP with CCS.

Initially, CSFP was considered as an agiographical finding, which had an incidence varied from 1 to 7% in patient with chest pain and non-obstructive coronary arteries [3, 6]. With further research, prof. Leone MC, et al. discovered that different from cardiac X syndrome (CXS), patients with CSFP tended to suffer rest chest pain and encountered a high incidence of acute coronary syndromes [18]. Moreover, CSFP is more common in young males and smokers, while CXS usually occurred in postmenopausal females [18]. So to distinguish CSFP from CXS, prof. Leone MC, et al. suggested that CSFP should be be regarded as a separate clinical entity called cardiac Y syndrome (CYS) [18]. Different from previous study, we included patients with CSFP and CCS, although only a low proposition of CSFP encountered CCS. We discovered that patients with CSFP tended to have different risk factors compared with CAD. Although CSFP may relate to cardiovascular risk factors such as hypertension, diabetes mellitus, dyslipidemia, and hyperuricemia. However, the potential risk factor and the independent predictors for CSFP in the literature remain debated. In this study, we found that CSFP was associated with hypertension and smoking. Further multivariate logistic regression analysis showed no correlation between CSFP and hypertension as well as smoking. We suggested that different from CAD, CSFP have an unique pathophysiology as well as risk factors. So it is of vital importance to explore the potential predictors of CSFP, therefore improve the management of these specific patients.

Although the exact pathogenesis of CSFP remains unclear, however, increasing evidences showed that CSFP was related to chronic inflammation [2–4], subclinical atherosclerosis [5], oxidative stress [4], and endothelial dysfunction [4, 6]. Prof Wang, et al. discovered that patients with CSFP had a higher level of systemic immune-inflammation index (SII) and SII was an independent predictor for CSFP [3]. Camsari et al. Suggested that atherosclerosis was the potential pathology for the development of CSFP [19]. Prof Wang, et al. also discovered that an elevated level of plasma thrombomodulin levels was an independent predictor for CSFP, which suggested that oxidative stress and endothelial dysfunction may participate in the occurrence and development of CSFP [4].

Uric acid has been suggested as a risk factor for atherosclerosis [9], an elevated level of uric acid is demonstrated to associate with endothelial dysfunction and increased chronic inflammatory response [11]. Hyperuricemia is related to the development of coronary atherosclerosis [20]. In patients with CAD, an increased level of plasma uric acid has been considered as a predictor for death [21]. Moreover, plasma uric acid was associated with the severity of coronary lesions defined by SYNTAX score [20]. Uric acid precipitated and generated monosodium urate crystals, bring in local inflammatory responses. Uric acid might also cause oxidative stress response, together with endothelial dysfunction and chronic inflammatory response [22], all of which could relate to the development of CSFP. Similar to previous study, we discovered that uric acid was associated with CSFP and was an independent predictor for CSFP [23]. As the most abundant protein in human body, the serum albumin < 35 g/L has been demonstrated to be a predictor for short term death as well as heart failure in ACS patients [24]. Chieh et al. [25], suggested that a lower plasma albumin level was associated with 1.5-year death in patients with CCS. Increasing evidences have suggested the serum albumin plays an important role in anti-inflammatory [13] and eliminating free radicals [14], which could bring in a protective effect for endothelium cell. Moreover, plasma albumin can maintain endothelial cell membrane stability and the balance of fluid across the capillary wall [26]. So a lower level of plasma albumin is related to the higher risk of atherosclerosis and poor prognosis, which further support the potential role of albumin for CSFP. In the process of inflammation, the synthesis of albumin decreased and the consumption increased [27], which in turn aggravated inflammatory response, resulting in a vicious cycle.

Recently, UAR has been suggested as a novel biomarker that reflects the overall inflammatory and oxidative status [11]. UAR combined the metabolic indicator, inflammation, and nutritional indices, whose role in cardiovascular disease has been investigated in very limited studies. Çakmak EÖ, et al., suggested that UAR was associated with the extent of CAD in NSTEMI [16]. A recent study suggested that UAR was related to the severity of CAD in patients with CCS [28]. An elevated level of UAR has also been proven as an independent predictor for new-onset atrial fibrillation in STEMI patients after primary PCI [29]. The more recent studies revealed that UAR was independently related to no-reflow in STEMI [30] and NSTEMI [31] patients undergoing PCI. Accumulating evidences have proven the predictive indicator for mortality in patients with ST segment elevation myocardial infarction (STEMI) [15], as well as non-ST segment elevation myocardial infarction (NSTEMI) [16], and unstable angina pectoris [17]. However, the relationship between UAR and CSFP has not been evaluated in the literature. Since the uric acid to albumin have the same pathophysiological basis with CSFP. So in this study, we aimed to explore the potential relationship between UAR and CSFP. We discovered that patients with CSFP had a higher level of UAR and an elevated level of UAR was an independent predictor for CSFP. Moreover, combined uric acid with albumin, UAR served a better predictive value for CSFP. We concluded the mechanism of UAR involved in CSFP as follows: (1) chronic inflammatory reaction; (2) oxidative stress; (3) local or systemic atherosclerosis relating to inflammatory reaction, oxidative stress and endothelial dysfunction. However, an increased level of UAR may result from the reduced level of albumin or an elevated level of uric acid. So an increased level of UAR might be a sign of malnutrition and hyper-catabolism. However, in this study, we excluded the patients with malnutrition and hyper-catabolism. So these results could not be extended to these populations. Nevertheless, as an easily acquired indicator, UAR may be a promising predictor for CSFP in CCS and perhaps a predictor for prognosis.

### Limitations

This study also had some limitations. First, this was a single-center study with a small sample size. We only included CSFP patients with CCS, so the result could not applied to other populations. Secondly, since the risk factors of CSFP remain unclear, despite our efforts to include additional factors, there may still be some residual covariates that could potentially affect the predictive value of UAR. Thirdly, although UAR could serve as a predictor for CSFP, the causal relationship between UAR and coronary slow flow phenomenon could not be determined. Fourthly, due to the limited number of patients in CSFP group, we didn’t perform a analysis between diabetic and non-diabetic patients, although they had a quite different vascular involvement due to the nature of the disease. Last but not the least, the clinical significance and outcome of UAR still require confirmation from future multicenter prospective studies.

### Future directions

In clinical practice, UAR could be used as a predictor CSFP in CCS patients. However, The role of UAR in the prediction of poor prognosis for CSFP patients remains unclear. Moreover, it is unclear whether CSFP could benefit from the uric acid lowering therapy or anti-inflammatory. In the future, large sample, prospective multi-center studies are required to explore the potential risk factors, the prognosis, as well as treatment in CSFP patients.

## Conclusion

UAR was an independent predictor for CSFP in patients with CCS.

## Data Availability

The datasets generated and analysed during the current study are not publicly available due to a further study of this area but are available from the corresponding author on reasonable request.

## Abbreviations

CSFP: Coronary slow flow phenomenon
CCS: Chronic coronary syndrome
UAR: Uric acid to albumin ratio
CAD: Coronary artery disease
TIMI: Thrombolysis in Myocardial Infarction
TFC: TIMI frame count
LAD: Left anterior descending
LCX: Left circumflex
RCA: Right coronary artery
ACEI: Angiotensin converting enzyme inhibitors
ARB: Angiotensin II receptor blocker
ARNI: Angiotensin receptor enkephalinase inhibitor
HDL-C: High-density lipoprotein cholesterol
LDL-C: Low-density lipoprotein cholesterol
